# Digital twin data-driven proactive job-shop scheduling strategy towards asymmetric manufacturing execution decision

**DOI:** 10.1038/s41598-022-05304-w

**Published:** 2022-01-28

**Authors:** Fuqiang Zhang, Junyan Bai, Dongyu Yang, Qiang Wang

**Affiliations:** 1 Key Laboratory of Road Construction Technology and Equipment of MOE, Chang’an University, Xi’an, 710064 Shaanxi China; 2 Institute of Smart Manufacturing Systems Engineering, Chang’an University, Xi’an, 710064 Shaanxi China; 3 China Electronic Product Reliability and Environmental Testing Research Institute, Guangzhou, 510610 China

**Keywords:** Mechanical engineering, Information technology

## Abstract

The information asymmetry phenomenon widely exists in production management decisions due to the latency of manufacturing data transmissions. Also, stochastic events on the physical production site will result in information asymmetry, which may lead to inconsistency between current execution and previous resource allocation plans. It is meaningful and important for developing an information model based on the Internet of Manufacturing Things to timely and actively adjust the scheduling strategy to meet the symmetry requirements of the production execution process. Based on the digital twin data collected from the workshop, a proactive job-shop scheduling strategy was discussed in this paper. Firstly, the mechanism for the influence of delayed local operations on makespan was deduced. Then, a framework for implementing the proactive job-shop scheduling strategy was proposed. Coordination point was used to determine the adjustment interval of local operations; right-shift rule with delay time constraints was used to adjust the unprocessed operation sequences on machines. Finally, the examples including 6*6 (6 jobs, 6 machines) and 20*40 (20 jobs, 40 machines) were presented to verify the effectiveness and scalability of the proposed method. It can be predicted that the proactive scheduling strategy provides the online decisions for the efficient and smooth execution of the digital twin-driven workshop production.

## Introduction

Due to the latency of manufacturing data transmissions and stochastic events on the physical production site, the information asymmetry phenomenon has widely existed in the production management decision process, which may lead to inconsistency between current execution and previous resource allocation plans. Proactive scheduling is an important part of maintaining the symmetry decision in workshops. Fierce market competition, personalized customer demand, and service-oriented production have prompted small and medium-sized enterprises to switch to a multi-variety, small-batch flexible production model^1,2^. In this case, enterprises must enhance their scheduling capabilities to shorten the product delivery cycles, increase equipment utilization, and reduce production costs to obtain greater benefits. Focusing on the traditional job-shop scheduling problems, the processing time for different jobs on the machine was estimated based on simulation software or practical experience. However, the uncertainty factors, such as the shortage of AGVs (Automated Guided Vehicles) or incorrect operation of workers, can lead to the actual processing time being inconsistent with the estimated theoretical processing time. In this situation, how to adjust the strategy in time and actively maintain the symmetry of the digital twin workshop is very important. It must be pointed out that the delay in makespan is an important macro index to evaluate the symmetry of the digital twin workshop. The delay in makespan *R* can be measured by the maximum completion time delay rate, which can be formulated as:

1 $$ R = \frac{{ms - ms_{0} }}{{ms_{0} }} \times 100\% $$

where *ms* denotes the actual makespan; *ms*_0_ denotes the theoretical makespan.

With the Internet of Manufacturing Things (IoMT) development, all kinds of sensors are used to monitor and control the production process in a digital twin workshop^3–6^. Among them, RFID (Radio Frequency Identification) technology for object recognition and tracking is the most mature technology in IoMT applications. Numerous industrial practices show that IoMT can provide us with the real-time tracking of production processes that suffer from unpredictable and hidden interference. In addition to these real-time on-site tracking information, the digital twin data are more extensive, including process data, such as process route planning; some predictive data, such as the processing time of unprocessed operations predicted by big data technology^7^. In the context of digital twin data, scheduling strategies must be determined to make the production run smoothly.

Based on the Gantt chart and digital twin data, a proactive job-shop scheduling strategy was discussed in this paper. When there are uncertain abnormal events, proactive scheduling can update or reschedule the initial scheme to minimize the impact of various interference factors on production performance. The main contributions of this paper are as follows:

- The definitions of critical path and critical operations were given. The mechanism for the influence of delayed local operations on the makespan was deduced.
- To maintain the initial scheduling scheme, the local adjustment rules for critical operations and non-critical operations were proposed.
- The case verifies the implementation framework of proactive scheduling, and the results show that the delay in makespan can be reduced to a certain extent.

The rest of this paper is organized as follows. “Brief review” section presents a brief review of proactive job-shop scheduling. Local operation delay impact on makespan is discussed in “Local operations delay impact on makespan” section. “Proactive scheduling strategy” section shows the proactive scheduling strategy and its implementation framework. In “Case study” section, the case study is presented. Conclusions are drawn in “Conclusion” section.

## Brief review

As a typical NP-hard combinatorial optimization problem, job-shop scheduling has always been a research hotspot in academia and industry^8^. Nowadays, industries are seeking models and methods that are not only able to provide efficient overall production performance, but also for reactive systems facing a growing set of unpredicted events^9^. The researchers have primarily focused on the stochastic resource-constrained scheduling problem. For example, Ghezail proposed a graphical representation of robustness to assist the decision-maker in understanding the consequences of possible perturbations^10^. Ryu incorporated the uncertainty in processing times and equipment availabilities into scheduling models, which were then transformed to multiparametric mixed-integer linear programming (mp-MILP) problems^11^. Jiang established a complex manufacturing network and a dynamic scheduling algorithm based on multi-layer network metrics were used to solve multi-resources and independent-task scheduling^12^. Sahin developed a multi-agent-based system for simultaneous scheduling of flexible machine groups and material handling systems working under a manufacturing dynamic environment^13^. Qin investigated the scheduling problem of a dynamic hybrid flow shop with uncertain processing time and an ant colony algorithm-based rescheduling approach was proposed^14^. Chen presented a solution algorithm consisting of a periodic scheduling policy and a modified genetic algorithm to solve the manufacturing synchronization in a hybrid flow shop with dynamic order arrivals^15^. The above stochastic scheduling is predictive scheduling when the manufacturing system is abnormally disturbed. Instead, proactive scheduling is a method to actively adjust production tasks based on the real-time manufacturing status of the shop floor. With the development of sensing technology, RFID and other technologies can be adapted to monitor and collect the state data of logistics in real time^16,17^. For example, Zhang presented a real-time information capturing and integration architecture, in which the Manufacturing Things such as operators, machines, pallets, materials, etc. can be embedded with sensors and interact with each other^6^. Qu investigated the typical production logistic execution operations and adopted system dynamics to design cost-effective solutions^18^. Zhong proposed a holistic Big Data approach to excavate frequent trajectory from massive RFID-enabled shopfloor logistics data^19^. Zhou established a production instruction service system through deploying RFID tags to smart workpieces^20^. Meanwhile, digital twin technology provides an infrastructure framework and related enabling technologies for implementing an intelligent workshop^5,21^. For example, Zhang adopted a five-dimension DT model to describe the machine availability prediction, disturbance detection and performance evaluation in a job-shop^22^. Liu presents a digital twin-based approach for rapid individualized designing of the hollow glass production line^23^. It can be seen from the above that digital twin data is the basis for constructing digital twin model; and how to combine these digital twin data to optimize production is the main research direction of proactive scheduling.

Therefore, this paper proposed a proactive scheduling strategy. The functional relationship between the delay in makespan and the actual completion time of each operation can be established. According to the coordination point of the given delay in makespan, its adjustment interval can be calculated. By adopting the right-shift strategy with the delay time constraints, the unprocessed sequence on the machine can reduce the delay in makespan.

## Local operations delay impact on makespan

### Critical operations

**Figure 1:**
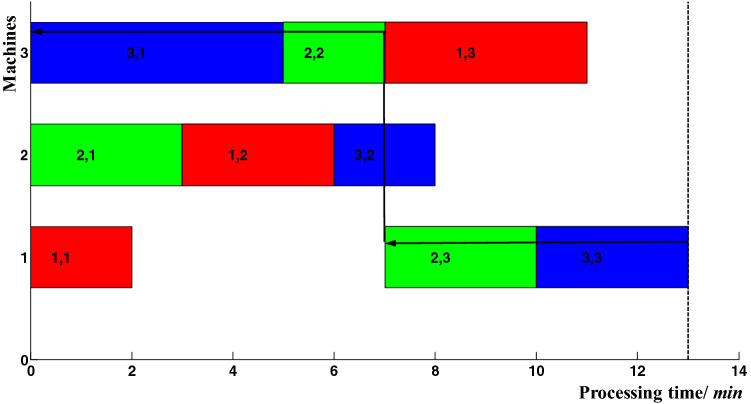
A simple example of a critical path.

Before analyzing the effect of local operation delay on makespan, it is essential to introduce the concepts of critical path and critical operations. Critical path refers to the longest path without slack time between different operations in the Gantt chart, which determines the makespan. It can be drawn from the last operation, searching forward by the means of "end to end" until the beginning of the Gantt chart. While these operations included in the critical path are defined as the critical operations. As shown in Fig. 1, there is one critical path with solid arrow *P*_3,1_ → *P*_2,2_ → *P*_2,3_* → P*_3,3_. The operations *P*_3,1_, *P*_2,2_, *P*_2,3*,*_ and *P*_3,3_ on the critical path, are regarded as critical operations; the rest of operations *P*_1,1_, *P*_2,1_, *P*_1,2_, and *P*_1,3_, are regarded as non-critical operations.

In Fig. 1, there is no slack time between the critical operations. When the actual completion time of a critical operation exceeds its theoretical completion time, all subsequent critical operations on the same critical path must be moved backward. Therefore, the delayed completion of critical operations can lead to a delay in makespan.

### Non-critical operations

**Figure 2:**
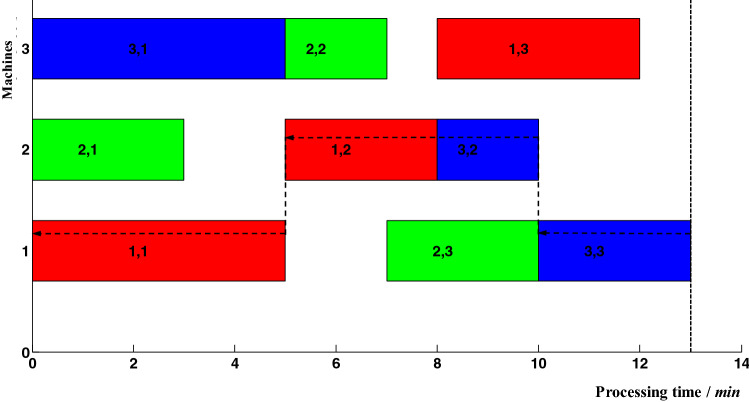
Delay analysis of non-critical operation *P*_1,1_.

In addition to the critical operations, the rest operations are called non-critical operations in the proactive job-shop scheduling. The delayed completion of non-critical operations can't immediately lead to a delay in makespan. Because each non-critical operation has a certain amount of delay buffer time, the sum of its theoretical completion time and delay buffer is the threshold level of its actual completion time. When the actual completion time is more than its threshold level, it can lead to a delay in makespan parameter R > 0. Figure 2 shows an example under the circumstance that the actual completion time of the non-critical operation *P*_1,1_ is equal to its threshold level.

Compared with Figs. 1 and 2, the theoretical completion time of non-critical operation *P*_1,1_ is 2. When its actual completion time is delayed to 5, a new critical path is formed as shown by the dotted arrow in Fig. 2. Therefore, the threshold level of the actual completion time of operation *P*_1,1_ is 5. Furthermore, the makespan can be affected by this critical path when its actual completion time is more than 5. Due to the cumulative effect of operation completion time delays, operation *P*_1,1_ can lead to delays in start-up time of operations *P*_1,2_ and *P*_3,2_, and further result in the delay in makespan. When the actual completion time of operation *P*_1_,_2_ is more than 8, it can lead to R > 0; when the actual completion time of operation *P*_3_,_2_ is more than 10, it can also lead to R > 0. Generally, the threshold level of the actual completion time of operation *P*_1_,_2_ is 8 and the delay buffer time is 2; the threshold level of the actual completion time of operation *P*_3_,_2_ is 10 and the delay buffer time is 2. The delayed completion of a local operation can result in the processing time delays of the operation itself and the start-up time delays of subsequent operations, or a combination of the above two cases. In either case, the delay in makespan is because the actual completion time is more than its threshold level.

### Delay impact on makespan

**Figure 3:**
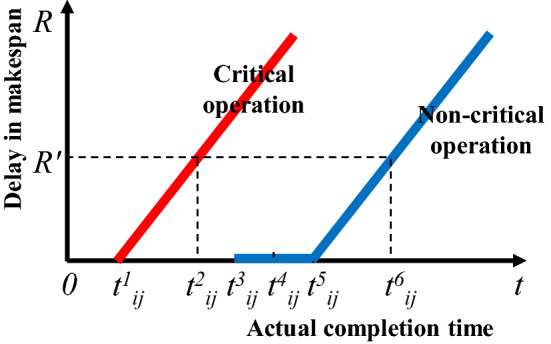
Influence curve between delay in makespan and actual completion time of critical/non-critical operations.

The above analysis about critical and non-critical operations shows that: (1) the threshold level of actual completion time of critical operations is the theoretical completion time. There is a strict proportional function relationship between the delay in makespan and the completion time delay of critical operations. (2) The completion time delay of non-critical operations can be viewed as a process that consumes its buffer time continuously, or it can be a process of transforming from non-critical operations to critical operations. (3) When the actual completion time of a non-critical operation reaches its threshold level, it can form a new critical path that affects the makespan. The influence curve between the delay in makespan *R* and the actual completion time of critical/non-critical operations is shown in Fig. 3.

For the critical operation *P*_*ij*_, *t*^1^_*ij*_ denotes the theoretical completion time and the threshold level of its actual completion time. For the non-critical operation *P*_*ij*_, *t*^3^_*ij,*_ and *t*^5^_*ij*_ denote its theoretical completion time and the threshold level of its actual completion time, respectively. It is noted that the length "*t*^3^_*ij*_-*t*^5^_*ij*_" denotes the delay buffer of non-critical operations, and *t*^4^_*ij*_ denotes start-up time of subsequent operation, *t*^3^_*ij*_ ≦ *t*^4^_*ij*_ ≦ *t*^5^_*ij*_. The value *t*^4^_*ij*_ is equal to the smaller between start-up time of subsequent job operation and a start-up time of subsequent machine operation for the non-critical operation. For example, the start-up time of subsequent job operation *P*_1,2_ and a start-up time of subsequent machine operation *P*_2,3_ for the non-critical operation *P*_1,1_ are 3 and 7, respectively. Here, *t*^4^_11_ for the non-critical operation *P*_1,1_ is set as 3, which means that actual completion time *t*_11_ ≦ 3 of operation *P*_1,1_ does not have any influence on the start-up time of other unprocessed operations. Assume that the delay in makespan corresponding to critical operation at time *t*^2^_*ij*_ or non-critical operation at time *t*^6^_*ij*_ is *R* = *R*′, and the corresponding makespan is *ms* = *ms*′. The influence curve of a non-critical operation has the same slope as the influence curve of a critical operation. Then,

2 $$ {\text{t}}^{2}_{{{\text{ij}}}} - {\text{t}}^{1}_{{{\text{ij}}}} = {\text{t}}^{6}_{{{\text{ij}}}} - {\text{t}}^{5}_{{{\text{ij}}}} $$

The equations of the above two influence curves can be formulated as:

3 $$ R = \frac{{R^{\prime}}}{{t_{ij}^{2} - t_{ij}^{1} }}\left( {t_{ij} - t_{ij}^{1} } \right),\quad t_{ij} > t_{ij}^{1} $$

4 $$ R = \left\{ {\begin{array}{*{20}l} {\frac{{R^{\prime } }}{{t_{ij}^{6} - t_{ij}^{5} }}} \hfill \\ {0,} \hfill \\ \end{array} } \right.\left( {t_{ij} - t_{ij}^{5} } \right),\begin{array}{*{20}l} {} \hfill & {t_{ij} > t_{ij}^{5} } \hfill \\ {} \hfill & {t_{ij}^{3} \le t_{ij} \le t_{ij}^{5} } \hfill \\ \end{array} $$

where *t*_*ij*_ denotes the actual completion time of operation *P*_*ij*_.

5 $$ {\text{t}}^{2}_{{{\text{ij}}}} - {\text{t}}^{1}_{{{\text{ij}}}} = {\text{ms}}^{\prime } - {\text{ms}}_{0} $$

6 $$ R^{\prime} = \frac{{ms^{\prime} - ms_{0} }}{{ms_{0} }} \times 100\% $$

The relationship between the delay in makespan and the actual completion time of the critical operation *P*_*ij*_ can be deduced from Eqs. (2)–(6):

7 $$ R = \frac{1}{{ms_{0} }}\left( {t_{ij} - t_{ij}^{1} } \right),\quad t_{ij} > t_{ij}^{1} $$

The relationship between the delay in makespan and the actual completion time of the non-critical operation *P*_*ij*_ and can be deduced:

8 $$ R = \left\{ {\begin{array}{*{20}l} {\frac{1}{{ms_{0} }}\left( {t_{ij} - t_{ij}^{5} } \right),} \hfill & {} \hfill \\ {0,} \hfill & {} \hfill \\ \end{array} \begin{array}{*{20}l} {t_{ij} > t_{ij}^{5} } \hfill \\ {t_{ij}^{3} \le t_{ij} \le t_{ij}^{5} } \hfill \\ \end{array} } \right. $$

Under the determined scheduling scheme, *ms*_0_, *t*^1^_*ij*_, *t*^3^_*ij*_, and *t*^5^_*ij*_ in Eqs. (7) and (8) are known parameters. Thus, as long as the actual completion time of the operation *P*_*ij*_ is determined, the delay in makespan can be calculated. Meanwhile, the actual completion time of corresponding operations can also be deduced based on a certain delay in makespan.

## Proactive scheduling strategy

Proactive scheduling is a dynamic scheduling method used to deal with the impact of uncertainty on the production process in a workshop. Its strategy is to adjust the start-up time and processing sequences of unprocessed operations based on the delay of the actual completion time of different operations relative to their estimated completion time, thereby avoiding large-scale rescheduling. The actual completion time of different operations can be collected in real time from the smart workshop supported by IoMT. In general, scheduling strategies are divided into right-shift adjustment and local adjustment. The right-shift adjustment is to move the entire start-up time of all the rest operations affected by the delayed operation backward without changing the initial job-shop scheduling sequence; local adjustment is a processing sequence adjustment strategy for individual unprocessed operations.

### Local adjustment

If the preceding operations of the unprocessed jobs have been finished, and the corresponding machine is idle, processing priority can be given to these unprocessed operations. Its purpose is to reduce the delay in makespan affected by delayed operation while maintaining the initial scheduling scheme as much as possible.

When the actual completion time of local operation is more than its threshold level, the delay in makespan can be R > 0. The proactive scheduling strategy is usually considered after the delay in makespan *R* exceeds a predetermined coordination point *R*_0_, that is, *R* > *R*_0_ (*R*_0_ > 0). Therefore, coordination point gives a range of adaptability to operation delays. If the unprocessed operations are adjusted in any case where R > 0, it can lead to chaotic scheduling results and waste of resources.

When the actual completion time of local operation exceeds the allowable range of *R*_0_, there are usually three possible results after implementing a proactive scheduling strategy. (1) Smaller delay in makespan; (2) the same delay in makespan; (3) more delay in makespan. The reason for the above three cases is related to the position of the moving operation in the Gantt chart.

### Local adjustment rules

To reveal the local adjustment rules, the concept of critical operation blocks was introduced. According to the definition of the critical path, there was no slack time between critical operations. Here, a critical operation block is defined as a set of critical operations that are allocated on the same machine without slack time between each other. The first block in the critical path is called the head block; the last block in the critical path is called the tail block; the rest blocks in the critical path are called middle blocks. The critical path shown in Fig. 1 contains two critical operation blocks, which are *P*_3,1_ and *P*_2,2_ on 3_rd_ machine; *P*_2,3_ and *P*_3,3_ on 1st machine. Since proactive scheduling is used to adjust the unprocessed operations, critical operation blocks must be re-searched based on the current processing time point. In the new critical path shown in Fig. 2, *P*_1,2_ and *P*_3,2_ on 2nd machine form the critical operation block. In proactive job-shop scheduling, the following rules can be observed:

1. Do not adjust the non-critical operations. Any sequence adjustment of the non-critical operations cannot reduce the delay in makespan.
2. For the head block in the critical path, its latter operation can be adjusted to reduce the delay in makespan.
3. For the middle blocks in the critical path, their preceding and latter operations can be adjusted to reduce the delay in makespan.
4. For the tail block in the critical path, its preceding operation can be adjusted to reduce the delay in makespan.
5. If there are abnormalities in many operations at the same time, they will be executed following the priority order. Critical operations are processed preferentially than non-critical operations; operations with larger delays are processed preferentially.

### Implementation framework

**Figure 4:**
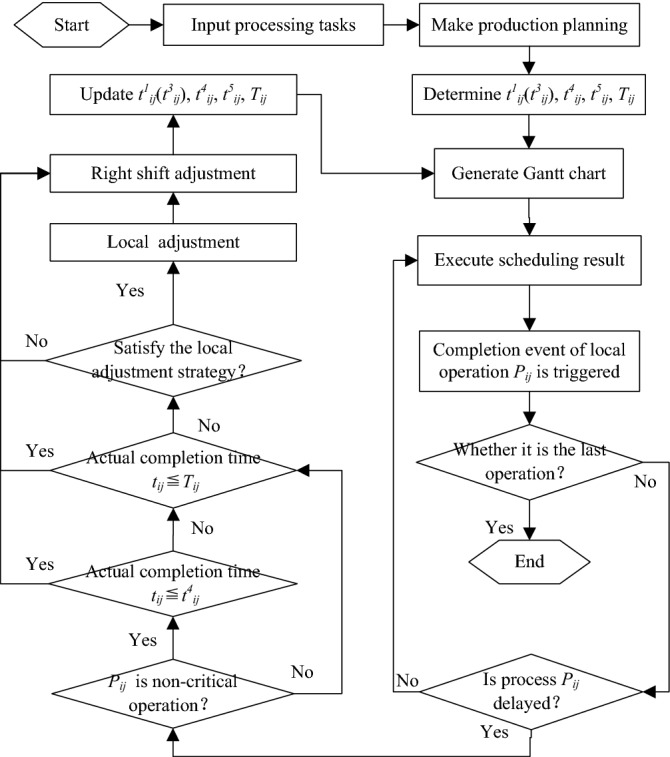
A framework for implementing proactive job-shop scheduling.

**Figure 5:**
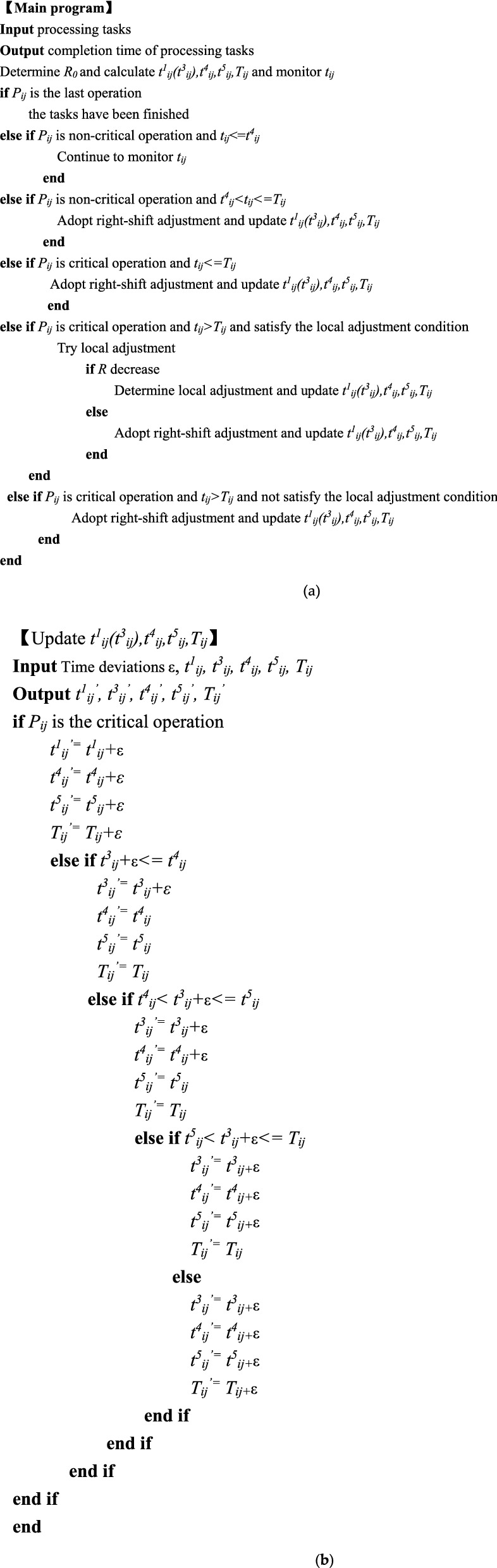
The pseudocode of implementing proactive job-shop scheduling. (**a**) The entire pseudocode of proactive job-shop scheduling; (**b**) The pseudocode of “Update *t*^1^_*ij*_(*t*^3^_*ij*_), *t*^4^_*ij*_, *t*^5^_*ij*_, *T*_*ij*_”.

The purpose of proactive job-shop scheduling is to try to reduce the delay in makespan *R* through right-shift adjustment and local adjustment. Figure 4 shows an implementation framework of proactive job-shop scheduling. Figure 5a shows the corresponding pseudocode of implementing proactive job-shop scheduling.

Detailed implementation steps are as follows:

*Step 1* Make production planning and generate a Gantt chart.

*Step 2* Determine the coordination point of the maximum completion time delay and calculate the relevant time parameters (*t*^1^_*ij*_, *t*^3^_*ij*_, *t*^4^_*ij*_, *t*^5^_*ij*_, *T*_*ij*_) of each operation.

*Step 3* Monitor the actual completion time of each operation in real time. If the current operation is the last operation, the tasks have been finished. Otherwise, continue to the next step.

*Step 4* If the current operation is a non-critical operation and its actual completion time is less than the start-up time of the next operation, it can be moved to Step3. Otherwise, continue to the next step.

*Step 5* If the actual completion time of the current operation is less than the corresponding completion time of the coordination point, the rest operation can be adjusted to the right and moved to Step1. At the same time, update *t*^1^_*ij*_(*t*^3^_*ij*_)*,t*^4^_*ij*_*,t*^5^_*ij*_*,T*_*ij*_. Otherwise, continue to the next step.

*Step 6* If there is an operation that can be partially adjusted and the maximum completion time delay is reduced after trial adjustment, the rest operation can be partially adjusted and moved to Step 1. At the same time, update *t*^1^_*ij*_(*t*^3^_*ij*_)*,t*^4^_*ij*_*,t*^5^_*ij*_*,T*_*ij*_. Otherwise, the rest operation can be adjusted rightly and moved to Step1.

It must be noted that in Step 2, *P*_*ij*_ denotes *j*_*th*_ operation of *i*_*th*_ job. *t*^1^_*ij*_ denotes the estimated theoretical completion time of critical operation; *t*^3^_*ij*_ denotes the theoretical completion time of non-critical operation; *t*^4^_*ij*_ denotes the start-up time of subsequent operation. The above three parameters can be extracted from the CAPP (Computer Aided Process Planning) database. *t*^5^_*ij*_ denotes the threshold level of the actual completion time of non-critical operation, which is the smaller of the start-up time of its job-back operation and machine-back operation. *T*_*ij*_ denotes the completion time of operation *P*_*ij*_ corresponding to the coordination point *R*_0_*.* For the critical operations, *T*_*ij*_ can be calculated by Eq. (7); for the non-critical operations, *T*_*ij*_ can be calculated by Eq. (8). *t*_*ij*_ denotes the actual completion time of operation *P*_*ij*_, which can be collected from RFID. In other steps, the pseudocode of “update *t*^1^_*ij*_(*t*^3^_*ij*_)*,t*^4^_*ij*_*,t*^5^_*ij*_*,T*_*ij*_” has been shown in Fig. 5b. The coordination point *R*_0_ = *R*_1_ of the delay in makespan of the initial scheduling scheme is determined according to the decision-makers’ demands. Assume that the theoretical makespan of the new scheme after *l* adjustment is *ms*_0_ = *ms*_*l*+1_, and coordination point of the delay in makespan is *R*_0_ = *R*_*l*+1_, then:

9 $$ R_{l + 1} = \left\{ {\begin{array}{*{20}l} {\frac{{R_{l} ms_{l} + ms_{l} - ms_{l + 1} }}{{ms_{l + 1} }},} \hfill & {ms_{l} \le ms_{l + 1} \le ms_{l} \left( {R_{l} + 1} \right)} \hfill \\ {0,} \hfill & {ms_{l + 1} > ms_{l} \left( {R_{l} + 1} \right)} \hfill \\ \end{array} } \right.,\quad l = 1,\;2,\;3,\; \ldots $$

## Case study

To verify the effectiveness and scalability of the proposed proactive scheduling strategy, classical scheduling cases including 6*6 (6 jobs, 6 machines) and 20*40 (20 jobs, 40 machines) were taken as examples to illustrate.

### Proactive scheduling in a 6*6 example

As shown in Table 1, there were 6 jobs; each job has six operations; machine and estimated theoretical processing time corresponding for each operation were given^24^.

**Table 1:** Machine tools (estimated machining time/min) corresponding to each operation.

Jobs	Operations					
	1	2	3	4	5	6
J_1_	M3(1)	M1(3)	M2(6)	M4(7)	M6(3)	M5(6)
J_2_	M2(8)	M3(5)	M5(10)	M6(10)	M1(10)	M4(4)
J_3_	M3(5)	M4(4)	M6(8)	M1(9)	M2(1)	M5(7)
J_4_	M2(5)	M1(5)	M3(5)	M4(3)	M5(8)	M6(9)
J_5_	M3(9)	M2(3)	M5(5)	M6(4)	M1(3)	M4(1)
J_6_	M2(3)	M4(3)	M6(9)	M1(10)	M5(4)	M3(1)

**Figure 6:**
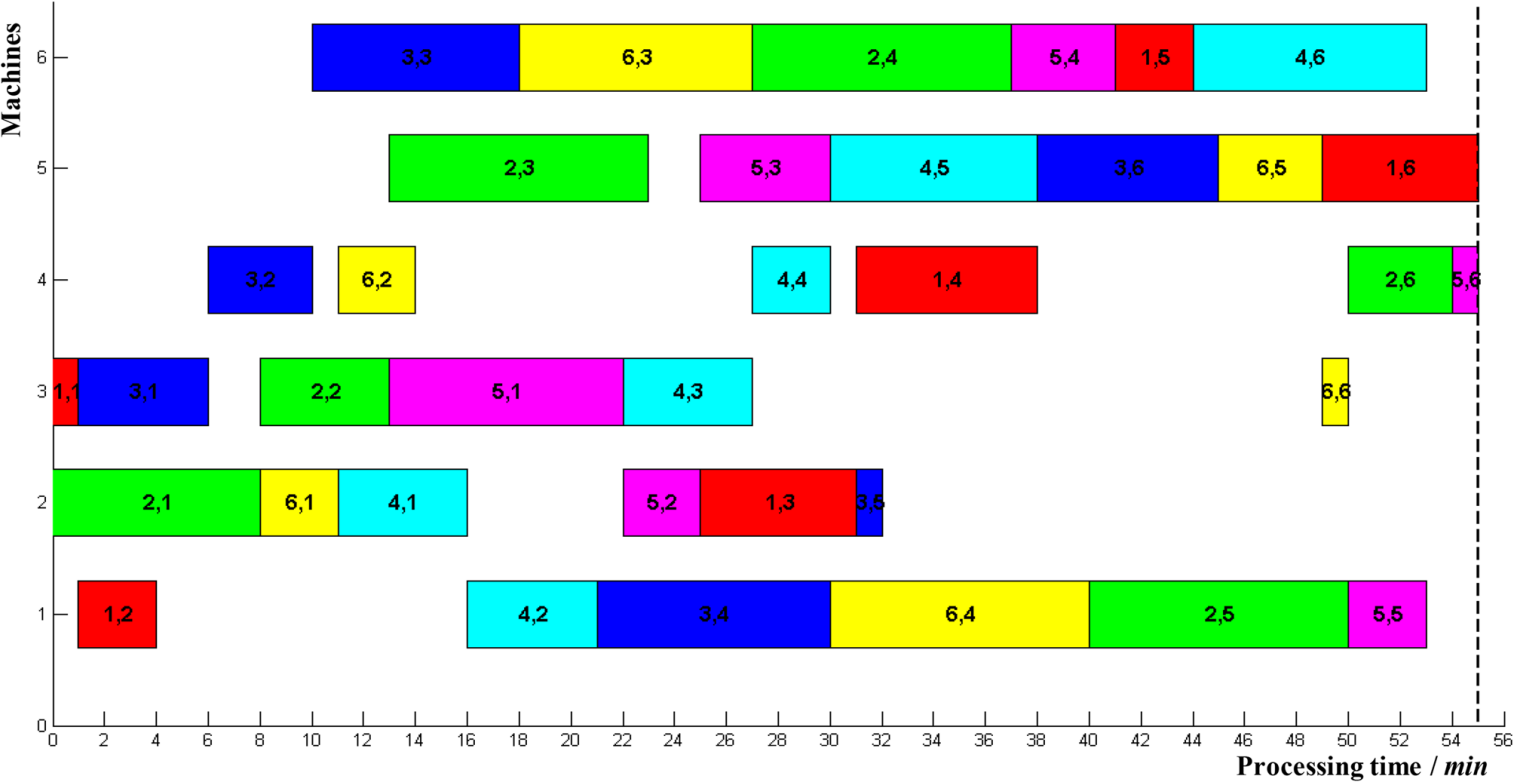
Gantt chart of 6*6 example.

At present, the optimal scheme of 6*6 example was 55, and a processing sequence in reference^25^ was selected. Figure 6 showed the corresponding Gantt chart, which was regarded as the initial scheduling scheme. There were two critical paths, including *P*_2,1_ → *P*_2,2_ → *P*_5,1_ → *P*_5,2_ → *P*_5,3_ → *P*_4,5_ → *P*_3,6_ → *P*_6,5_ → *P*_1,6_ and *P*_2,1_ → *P*_6,1_ → *P*_4,1_ → *P*_4,2_ → *P*_3,4_ → *P*_6,4_ → *P*_2,5_ → *P*_2,6_ → *P*_5,6_. Therefore, *P*_2,1_, *P*_2,2_, *P*_5,1_, *P*_5,2_, *P*_5,3_, *P*_4,3_, *P*_4,4_. *P*_4,5_, *P*_3,6_, *P*_6,5_, *P*_1,6_, *P*_6,1_, *P*_4,1_, *P*_4,2_, *P*_3,4_, *P*_6,4_, *P*_2,5_, *P*_2,6_, *P*_5,6_ were critical operations; the rest were non-critical operations. The theoretical completion time *t*^1^_*ij*_ (*t*^3^_*ij*_) of operation *P*_*ij*_ and the start-up time of subsequent non-critical operations were shown in Table 2.

**Table 2:** Theoretical completion time of each operation and start-up time of subsequent non-critical operations.

P_ij_	1,1	1,2	1,3	1,4	1,5	1,6	2,1	2,2	2,3	2,4	2,5	2,6
t^1^_ij_ (t^3^_ij_)	1	4	31	38	44	55	8	13	23	37	50	54
t^4^_ij_	1	16	31	41	44	–	–	–	25	37	–	–

P_ij_	3,1	3,2	3,3	3,4	3,5	3,6	4,1	4,2	4,3	4,4	4,5	4,6
t^1^_ij_ (t^3^_ij_)	6	10	18	30	32	45	16	21	27	30	38	53
t^4^_ij_	6	10	18	–	38	–	–	–	–	–	–	55

P_ij_	5,1	5,2	5,3	5,4	5,5	5,6	6,1	6,2	6,3	6,4	6,5	6,6
t^1^_ij_ (t^3^_ij_)	22	25	30	41	53	55	11	14	27	40	49	50
t^4^_ij_	–	–	–	41	54	–	–	18	27	–	–	55

Deviations in completion time for different local operations may lead to the delay in makespan. The deduced delays in makespan were listed in Tables 3, 4, 5, 6, 7 and 8. It is noted that time deviation *ε* of local operations was actual completion time minus their theoretical completion time.

**Table 3:** The delay in makespan due to operation delay of 1st job.

Time deviations ε of local operation (min)	Delay in makespan (%)					
	1,1	1,2	1,3	1,4	1,5	1,6
0	0	0	0	0	0	0
1	0	0	0	0	0	1.8
2	0	0	0	0	0	3.6
3	1.8	0	0	0	1.8	5.4
4	3.6	0	0	0	3.6	7.2
5	5.4	0	0	0	5.4	9.0
6	7.2	0	1.8	1.8	7.2	10.8
7	9.0	0	3.6	3.6	9.0	12.6
8	10.8	0	5.4	5.4	10.8	14.4
9	12.6	0	7.2	7.2	12.6	16.2
10	14.4	0	9.0	9.0	14.4	18.0
11	16.2	0	10.8	10.8	16.2	19.8
12	18.0	0	12.6	12.6	18.0	21.6
13	19.8	1.8	14.4	14.4	19.8	23.4
14	21.6	3.6	16.2	16.2	21.6	25.2

**Table 4:** The delay in makespan due to operation delay of 2nd job.

Time deviations ε of local operation (min)	Delay in makespan (%)					
	2,1	2,2	2,3	2,4	2,5	2,6
0	0	0	0	0	0	0
1	1.8	1.8	0	0	1.8	1.8
2	3.6	3.6	0	0	3.6	3.6
3	5.4	5.4	1.8	1.8	5.4	5.4
4	7.2	7.2	3.6	3.6	7.2	7.2
5	9.0	9.0	5.4	5.4	9.0	9.0
6	10.8	10.8	7.2	7.2	10.8	10.8
7	12.6	12.6	9.0	9.0	12.6	12.6
8	14.4	14.4	10.8	10.8	14.4	14.4
9	16.2	16.2	12.6	12.6	16.2	16.2
10	18.0	18.0	14.4	14.4	18.0	18.0
11	19.8	19.8	16.2	16.2	19.8	19.8
12	21.6	21.6	18.0	18.0	21.6	21.6
13	23.4	23.4	19.8	19.8	23.4	23.4
14	25.2	25.2	21.6	21.6	25.2	25.2

**Table 5:** The delay in makespan due to operation delay of 3th job.

Time deviations ε of local operation (min)	Delay in makespan (%)					
	3,1	3,2	3,3	3,4	3,5	3,6
0	0	0	0	0	0	0
1	0	0	0	1.8	0	1.8
2	0	0	0	3.6	0	3.6
3	1.8	1.8	1.8	5.4	0	5.4
4	3.6	3.6	3.6	7.2	0	7.2
5	5.4	5.4	5.4	9.0	0	9.0
6	7.2	7.2	7.2	10.8	0	10.8
7	9.0	9.0	9.0	12.6	1.8	12.6
8	10.8	10.8	10.8	14.4	3.6	14.4
9	12.6	12.6	12.6	16.2	5.4	16.2
10	14.4	14.4	14.4	18.0	7.2	18.0
11	16.2	16.2	16.2	19.8	9.0	19.8
12	18.0	18.0	18.0	21.6	10.8	21.6
13	19.8	19.8	19.8	23.4	12.6	23.4
14	21.6	21.6	21.6	25.2	14.4	25.2

**Table 6:** The delay in makespan due to operation delay of 4th job.

Time deviations ε of local operation (min)	Delay in makespan (%)					
	4,1	4,2	4,3	4,4	4,5	4,6
0	0	0	0	0	0	0
1	1.8	1.8	1.8	1.8	1.8	0
2	3.6	3.6	3.6	3.6	3.6	0
3	5.4	5.4	5.4	5.4	5.4	1.8
4	7.2	7.2	7.2	7.2	7.2	3.6
5	9.0	9.0	9.0	9.0	9.0	5.4
6	10.8	10.8	10.8	10.8	10.8	7.2
7	12.6	12.6	12.6	12.6	12.6	9.0
8	14.4	14.4	14.4	14.4	14.4	10.8
9	16.2	16.2	16.2	16.2	16.2	12.6
10	18.0	18.0	18.0	18.0	18.0	14.4
11	19.8	19.8	19.8	19.8	19.8	16.2
12	21.6	21.6	21.6	21.6	21.6	18.0
13	23.4	23.4	23.4	23.4	23.4	19.8
14	25.2	25.2	25.2	25.2	25.2	21.6

**Table 7:** The delay in makespan due to operation delay of 5th job.

Time deviations ε of local operation (min)	Delay in makespan (%)					
	5,1	5,2	5,3	5,4	5,5	5,6
0	0	0	0	0	0	0
1	1.8	1.8	1.8	0	0	1.8
2	3.6	3.6	3.6	0	1.8	3.6
3	5.4	5.4	5.4	1.8	3.6	5.4
4	7.2	7.2	7.2	3.6	5.4	7.2
5	9.0	9.0	9.0	5.4	7.2	9.0
6	10.8	10.8	10.8	7.2	9.0	10.8
7	12.6	12.6	12.6	9.0	10.8	12.6
8	14.4	14.4	14.4	10.8	12.6	14.4
9	16.2	16.2	16.2	12.6	14.4	16.2
10	18.0	18.0	18.0	14.4	16.2	18.0
11	19.8	19.8	19.8	16.2	18.0	19.8
12	21.6	21.6	21.6	18.0	19.8	21.6
13	23.4	23.4	23.4	19.8	21.6	23.4
14	25.2	25.2	25.2	21.6	23.4	25.2

**Table 8:** The delay in makespan due to operation delay of 6th job.

Time deviations ε of local operation (min)	Delay in makespan (%)					
	6,1	6,2	6,3	6,4	6,5	6,6
0	0	0	0	0	0	0
1	1.8	0	0	1.8	1.8	0
2	3.6	0	0	3.6	3.6	0
3	5.4	0	1.8	5.4	5.4	0
4	7.2	0	3.6	7.2	7.2	0
5	9.0	0	5.4	9.0	9.0	0
6	10.8	0	7.2	10.8	10.8	1.8
7	12.6	1.8	9.0	12.6	12.6	3.6
8	14.4	3.6	10.8	14.4	14.4	5.4
9	16.2	5.4	12.6	16.2	16.2	7.2
10	18.0	7.2	14.4	18.0	18.0	9.0
11	19.8	9.0	16.2	19.8	19.8	10.8
12	21.6	10.8	18.0	21.6	21.6	12.6
13	23.4	12.6	19.8	23.4	23.4	14.4
14	25.2	14.4	21.6	25.2	25.2	16.2

In Tables 3, 4, 5, 6, 7 and 8, the sum of maximum time deviations *ε* corresponding to "0" value of each operation and its theoretical completion time *t*^1^_*ij*_ (*t*^3^_*ij*_) was the threshold level *t*^1^_*ij*_ (*t*^5^_*ij*_) of its actual completion time. For example, maximum time deviations *ε* corresponding to “0” value of *P*_6,2_ was 6. According to Table 2, its theoretical completion time was 14. Then, its threshold level of actual completion time was 20. Table 9 lists the threshold levels for the actual completion time of each operation under the initial scheduling scheme.

**Table 9:** Threshold level of different operations.

P_ij_	1,1	1,2	1,3	1,4	1,5	1,6	2,1	2,2	2,3	2,4	2,5	2,6
t^5^_ij_	3	16	36	43	46	55	8	13	25	39	50	54

P_ij_	3,1	3,2	3,3	3,4	3,5	3,6	4,1	4,2	4,3	4,4	4,5	4,6
t^5^_ij_	8	12	20	30	38	45	16	21	27	30	38	55

P_ij_	5,1	5,2	5,3	5,4	5,5	5,6	6,1	6,2	6,3	6,4	6,5	6,6
t^5^_ij_	22	25	30	43	54	55	11	20	29	40	49	55

**Figure 7:**
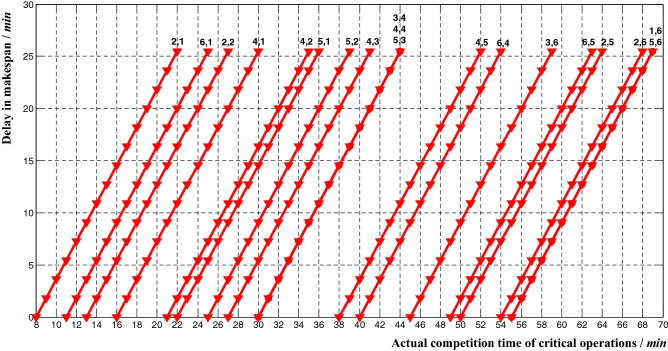
The relationship between delay in makespan and actual completion time of critical operations.

The theoretical makespan of 6*6 example was *ms*_1_ = 55, and the relationship between the actual completion time of critical operation and delay in makespan can be deduced from Eq. (7):

10 $$ R = \frac{1}{55}\left( {t_{ij} - t_{ij}^{1} } \right),\quad t_{ij} > t_{ij}^{1} $$

where *t*^1^_*ij*_ corresponds to the theoretical completion time of critical operation in Table 9. The corresponding influence curve was shown in Fig. 7.

**Figure 8:**
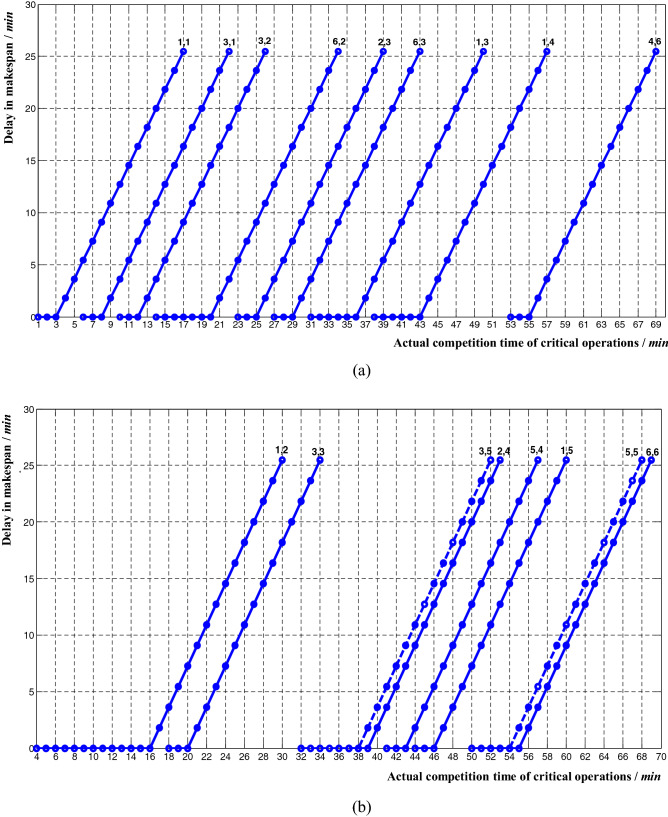
The relationship between delay in makespan and actual completion time of non-critical operations. (**a**) Non-critical operations *P*_11_, *P*_31_, *P*_32_, *P*_62_, *P*_23_, *P*_63_, *P*_13_, *P*_14_, *P*_46_; (**b**) Non-critical operations *P*_12_, *P*_33_, *P*_35_, *P*_24_, *P*_54_, *P*_15_, *P*_55_, *P*_66_.

From (8), the relationship between delay in makespan and actual completion time of non-critical operations can be deduced:

11 $$ R = \left\{ {\begin{array}{*{20}l} {\frac{1}{55}\left( {t_{ij} - t_{ij}^{5} } \right),} \hfill & {t_{ij} > t_{ij}^{5} } \hfill \\ {0,} \hfill & {t_{ij}^{3} \le t_{ij} \le t_{ij}^{5} } \hfill \\ \end{array} } \right. $$

where, *t*^3^_*ij*_ corresponds to the theoretical completion time of the non-critical operations in Table 2; *t*^5^_*ij*_ corresponds to a threshold level of actual completion time of non-critical operations in Table 9. The corresponding influence curve was shown in Fig. 8.

As shown in Figs. 7 and 8, there was a linear relationship between the delay in makespan and the actual completion time of critical and non-critical operations. The difference was that the non-critical operations had a certain amount of delay buffer time, which was consistent with the theoretical analysis results of the methods mentioned above.

Assume that the coordination point of delay in makespan determined by the initial scheduling scheme was *R*_1_ = 10%^26^. According to (10) and (11), the completion time *T*_*ij*_ of each operation corresponding to the coordination point can be calculated in Table 10. By monitoring the actual completion time *t*_*ij*_ of each operation and comparing it with the data in Tables 2 and 10, the corresponding adjustment decision can be made.

**Table 10:** Corresponding completion time *T*_*ij*_ of different operations with coordination point *R*_0_ = 10%.

P_ij_	1,1	1,2	1,3	1,4	1,5	1,6	2,1	2,2	2,3	2,4	2,5	2,6
T_ij_	8.56	21.56	41.56	48.56	51.56	60.56	13.56	18.56	30.56	44.56	55.56	59.56

P_ij_	3,1	3,2	3,3	3,4	3,5	3,6	4,1	4,2	4,3	4,4	4,5	4,6
T_ij_	13.56	17.56	25.56	35.56	43.56	50.56	21.56	26.56	32.56	35.56	43.56	50.56

P_ij_	5,1	5,2	5,3	5,4	5,5	5,6	6,1	6,2	6,3	6,4	6,5	6,6
T_ij_	27.56	30.56	35.56	48.56	59.56	60.56	16.56	25.56	34.56	45.56	54.56	60.56

**Figure 9:**
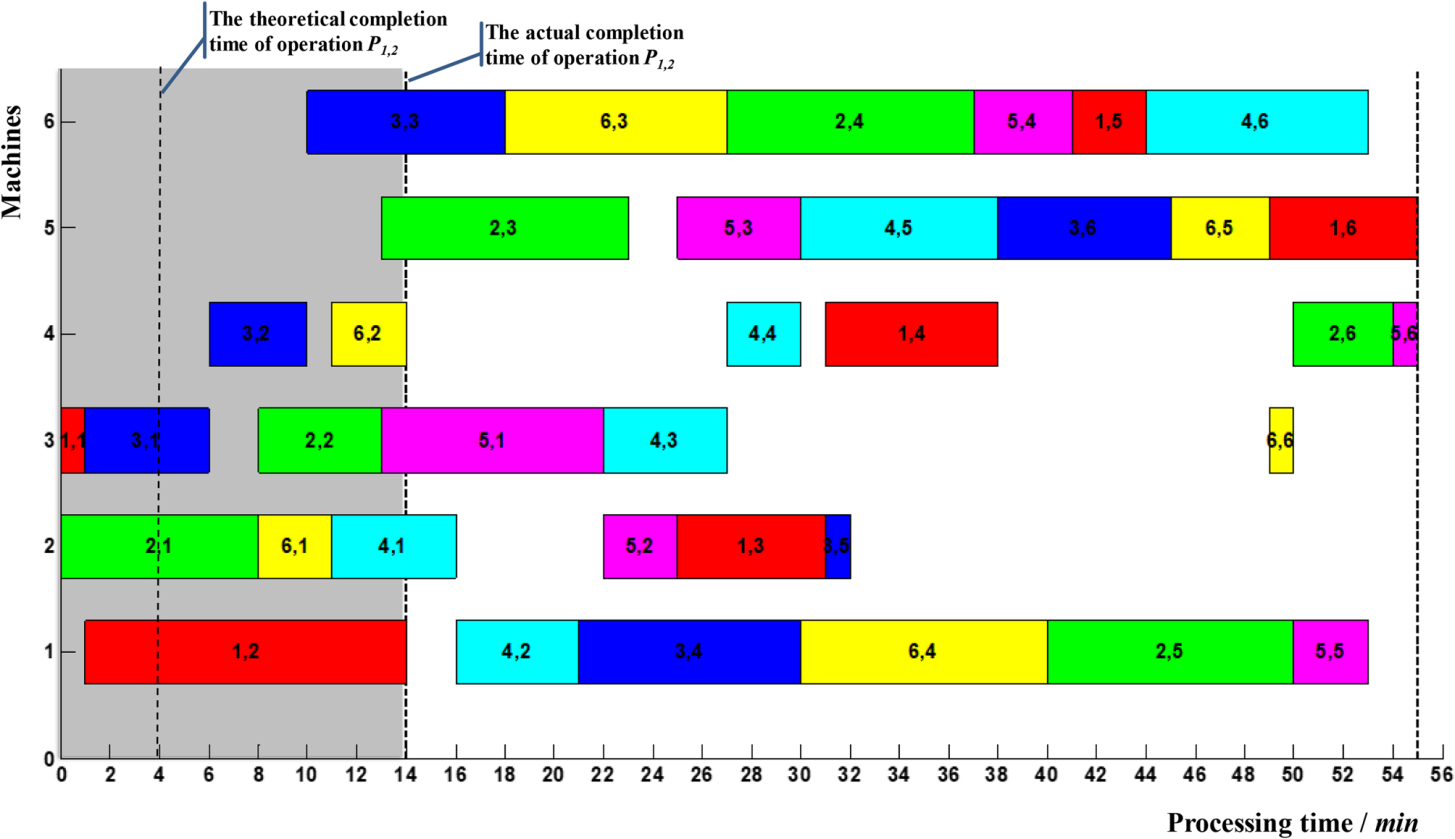
Delayed completion for non-critical operation *P*_1,2_.

Suppose the delayed completion of non-critical operation *P*_1,2_ was monitored at time 14, ie *t*_12_ = 14, as shown in Fig. 9. It is known from Table 2 that *t*^4^_12_ = 16, then *t*_12_ < *t*^4^_12_. The delayed completion of operation *P*_1,2_ has no effect on other operations, so the initial scheme continued.

**Figure 10:**
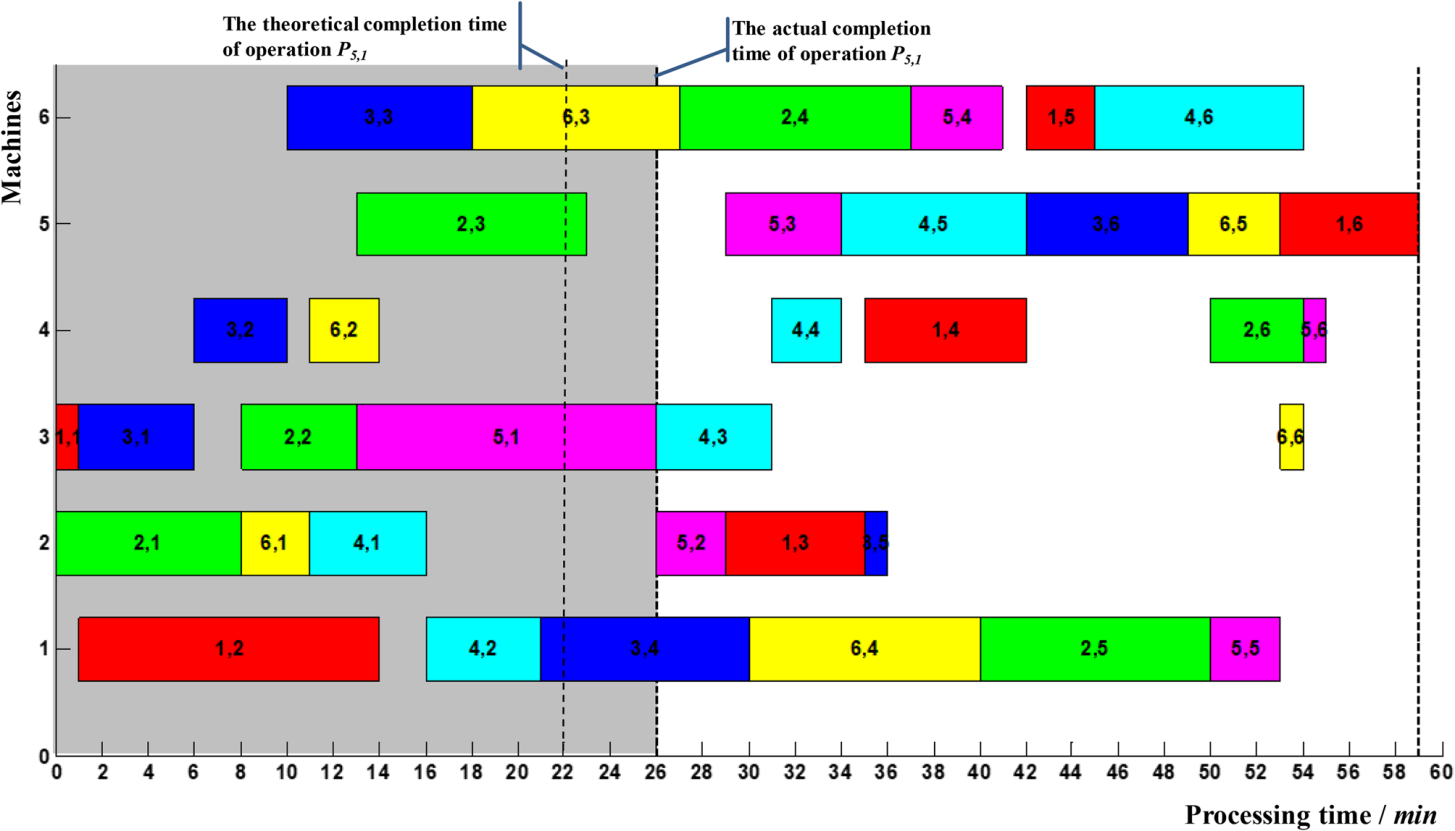
Delayed completion for critical operation *P*_5,1_.

Suppose the delayed completion of critical operation *P*_5,1_ was monitored at time 26, ie *t*_51_ = 26, as shown in Fig. 10. It is known from Table 10 that *T*_51_ = 27.56, then *t*_51_ < *T*_51_. The delayed completion of operation *P*_5,1_ did not cause the delay in makespan to exceed the coordination point. So, it was essential to make a right-shift adjustment for the relevant affected operations *P*_5,2_, *P*_1,3_, *P*_3,5_, *P*_4,3_, *P*_6,6_, *P*_4,4_, *P*_1,4_, *P*_5,3_, *P*_4,5_, *P*_3,6_, *P*_6,5_, *P*_1,6_, *P*_1,5_, *P*_4,6_.

After adopting the right-shift adjustment strategy, the makespan was *ms*_2_ = 59, and the coordination point calculated by (9) was *R*_2_ = 2.5%. The delay in makespan *R* of the rest operations can be formulated as follows:

12 $$ R = \frac{1}{59}\left( {t_{ij} - t_{ij}^{1} } \right),\quad t_{ij} > t_{ij}^{1} $$

13 $$ R = \left\{ {\begin{array}{*{20}l} {\frac{1}{59}\left( {t_{ij} - t_{ij}^{5} } \right),} \hfill & {t_{ij} > t_{ij}^{5} } \hfill \\ {0,} \hfill & {t_{ij}^{3} \le t_{ij} \le t_{ij}^{5} } \hfill \\ \end{array} } \right. $$

The calculated time parameters were shown in Table 11.

**Table 11:** Time parameters of the rest of operations after one adjustment.

P_ij_	t^1^_ij_	t^3^_ij_	t^4^_ij_	t^5^_ij_	T_ij_
3,4	–	30	30	34	35.47
6,4	–	40	40	44	45.47
2,5	–	50	50	54	55.47
5,5	–	53	54	58	59.47
5,2	29	–	–	–	30.47
1,3	–	35	35	40	41.47
3,5	–	36	42	42	43.47
4,3	31	–	–	–	32.47
6,6	–	54	59	59	60.47
4,4	34	–	–	–	35.47
1,4	–	42	42	47	48.47
2,6	–	54	54	58	59.47
5,6	–	55	59	59	60.47
5,3	34	–	–	–	35.47
4,5	42	–	–	–	43.47
3,6	49	–	–	–	50.47
6,5	53	–	–	–	54.47
1,6	59	–	–	–	60.47
6,3	–	27	27	33	34.47
2,4	–	37	37	43	44.47
5,4	–	41	42	47	48.47
1,5	–	45	45	50	51.47
4,6	–	54	59	59	60.47

**Figure 11:**
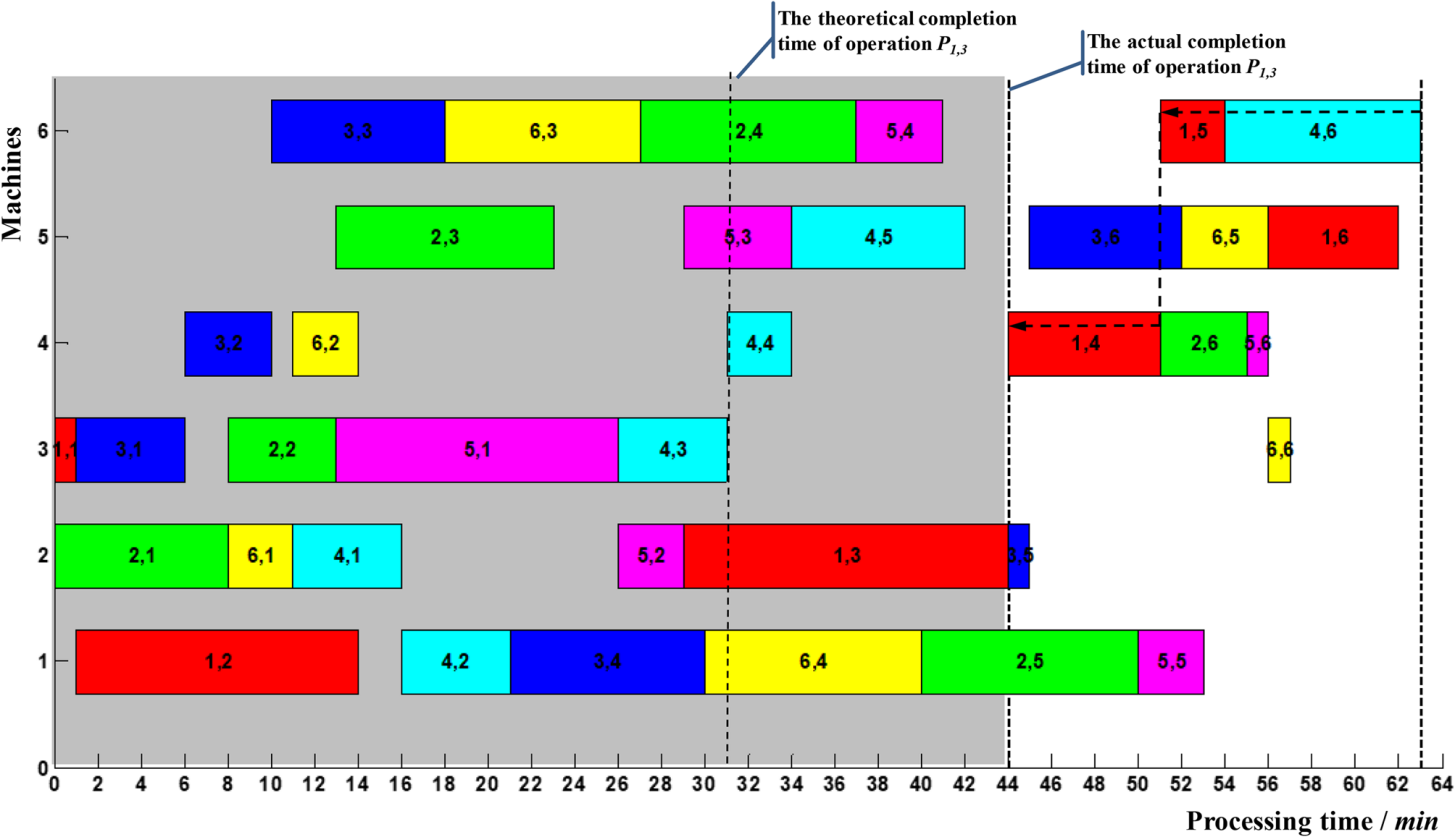
Delayed completion for non-critical operation *P*_1,3_.

**Figure 12:**
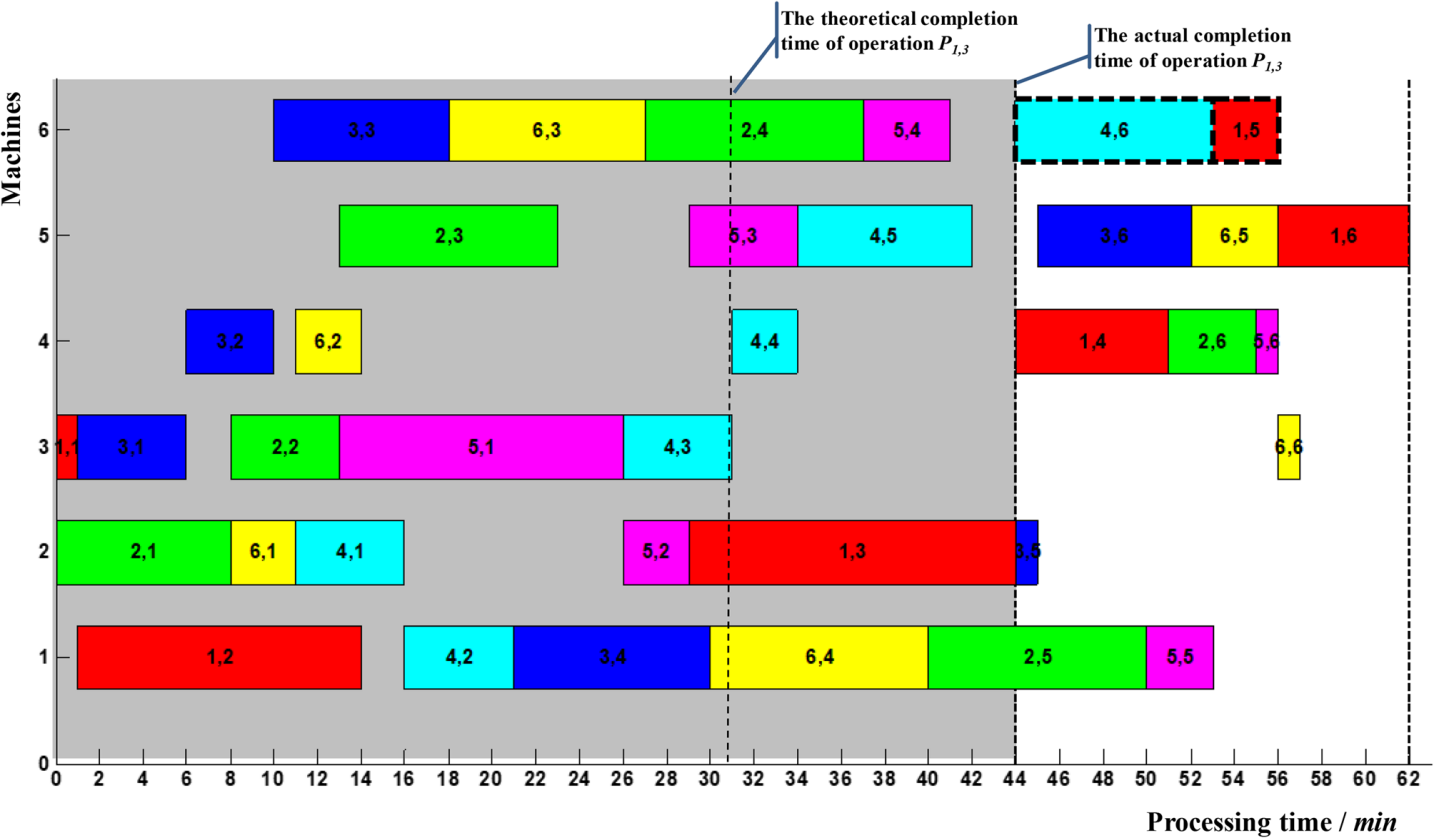
Local adjustment of operation *P*_4,6_.

Suppose the delayed completion of non-critical operation *P*_1,3_ was monitored at time 44, ie *t*_13_ = 44, as shown in Fig. 11. It is known from Table 11, *T*_13_ = 41.47. T_13_ > T_13_, and the corresponding delay in makespan *R* = 6.8% > 2.5%. The start-up time and processing sequences of the rest operations must be adjusted. In Fig. 11, the delayed completion of operation *P*_1,3_ formed a new critical path denoted by the dashed arrow. Operation *P*_1,4_ made up the first block; operations *P*_1,5_ and *P*_4,6_ made up the tail block. At present, the preceding operation *P*_4,5_ of operation *P*_4,6_ had been finished, and 6_th_ machine was idle. Therefore, operations *P*_4,6_ and operations *P*_1,5_ can be adjusted. The updated Gantt chart was shown in Fig. 12.

It can be seen from Fig. 12 that after the local adjustment of operation *P*_4,6_, the makespan was shortened from 63 to 62, and the delay in makespan *R* was reduced from 6.8% to 5.1%. According to (9), *R*_3_ = 0 can be calculated. The delay in makespan *R* of the rest operations can be formulated as follows:

14 $$ R = \frac{1}{62}\left( {t_{ij} - t_{ij}^{1} } \right),\quad t_{ij} > t_{ij}^{1} $$

15 $$ R = \left\{ {\begin{array}{*{20}l} {\frac{1}{62}\left( {t_{ij} - t_{ij}^{5} } \right),} \hfill & {t_{ij} > t_{ij}^{5} } \hfill \\ {0,} \hfill & {t_{ij}^{3} \le t_{ij} \le t_{ij}^{5} } \hfill \\ \end{array} } \right. $$

The calculated time parameters are shown in Table 12.

**Table 12:** Time parameters of the rest operations after two adjustment.

P_ij_	t^1^_ij_	t^3^_ij_	t^4^_ij_	t^5^_ij_	T_ij_
2,5	–	50	50	57	57.00
5,5	–	53	55	61	61.00
3,5	45	–	–	–	45.00
6,6	–	57	62	62	62.00
1,4	–	51	51	53	53.00
2,6	–	55	55	61	61.00
5,6	–	56	62	62	62.00
3,6	52	–	–	–	52.00
6,5	56	–	–	–	56.00
1,6	62	–	–	–	62.00
4,6	53	–	–	–	53.00
1,5	56	–	–	–	56.00

According to updated scheduling results, the rest 12 operations were processed until the last operation *P*_1,6_ was finished at time 62.

It must be noted that the computational cost of proactive scheduling was determined with the delay of each operation and the number of the rest unprocessed operations. The purpose of proactive scheduling was to try to reduce the entire completion time as much as possible through the fine adjustment of the local operation. In the example of 6*6, it was found that the calculation time did not exceed 10 s.

### Proactive scheduling in a 20*40 example

**Figure 13:**
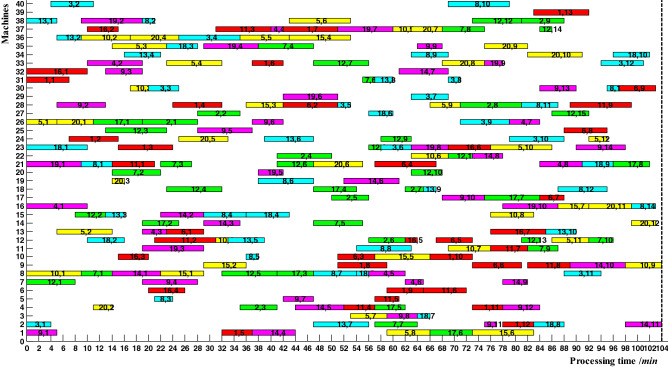
Gantt chart of 20*40 example.

In this example, there were 20 jobs and 40 machines. Table 13 showed the machines and the processing time corresponding to partial operations. At present, the optimal scheme of 20*40 example was 104, and its Gantt chart was shown in Fig. 13. According to the Gantt chart and coordination point, the time parameters corresponding to each operation can be calculated, which provided the comparison criteria for the collected actual completion time of different operations.

**Table 13:** Machines and processing time of partial operations for 20*40 example.

Jobs	Operations				
	1	2	3	…	8
*J*_1_	M31(7)	M24(8)	M23(9)	…	M39(9)
*J*_2_	M26(9)	M27(7)	M4(6)	…	M38(7)
*J*_3_	M2(4)	M40(7)	M30(5)	…	M33(7)
…	…	…	…	…	…
*J*_20_	M26(6)	M4(3)	M19(2)	…	M14(4)

**Figure 14:**
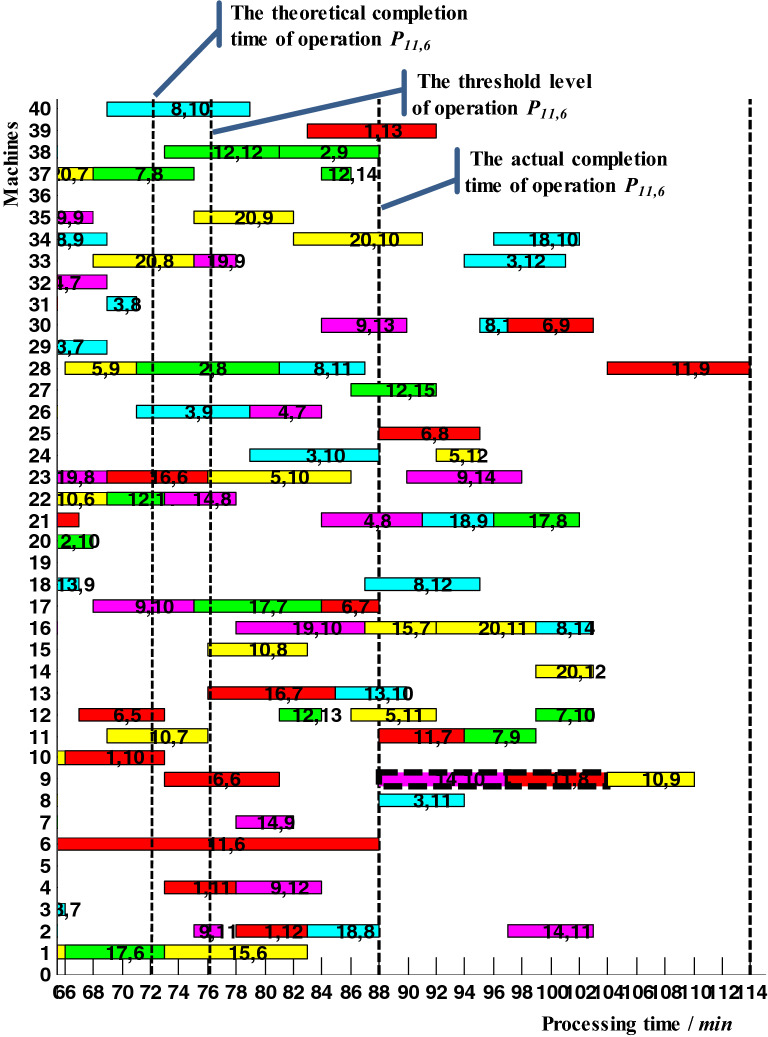
The updated Gantt chart after adopting local adjustment between operation *P*_14,10_ and operation *P*_11,8_.

Taking operation *P*_11,6_ as a case to verify the proactive scheduling strategy. *P*_11,6_ was a non-critical operation and its theoretical completion time *t*^1^_11,6_ was 72; the start-up time *t*^4^_11,6_ of its subsequent operation *P*_11,7_ was 76; the threshold level *t*^5^_11,6_ of its actual completion time was 76. Triggered by a monitoring event, the actual completion time of operation *P*_11,6_ was 88. In the production execution process, the actual completion time collected for operation *P*_11,6_ was 88, which was more than the threshold level *t*^5^_11,6_. Under these circumstances, the total completion time was delayed to 120. Here, local adjustment was adopted to reduce the delay in makespan. As shown in Fig. 14, *P*_14,10_ was adjusted to *P*_11,8_, and the corresponding total completion time was 114. Therefore, the time parameters of the rest 21 operations must be updated. According to the updated scheduling scheme, the whole production continued until the last operation *P*_11,9_ was finished at time 114.

## Conclusion

This paper proposes a proactive job-shop scheduling strategy driven by digital twin data to address the information asymmetry phenomenon caused by the latency of manufacturing data transmissions and stochastic events on the physical production site. By monitoring the completion time of different operations, the delay in makespan can be reduced by adjusting the start-up time and processing sequence of the unprocessed operations.

1. Aiming at the critical and non-critical operations, the influence mechanism of local operation delay in makespan was revealed. It is found that there was a linear relationship between the delay in makespan and the actual completion time of local operation, and its function formula was deduced.
2. To reduce the delay in makespan, local adjustment rules for unprocessed operations were given. Based on the coordination point, the corresponding time parameters (*t*^1^_*ij*_, *t*^3^_*ij*_, *t*^4^_*ij*_, *t*^5^_*ij*_, *T*_*ij*_) were calculated and compared with the actual completion time t_*ij*_ of local operation *P*_*ij*_. The local adjustment and right-shift strategy for the delayed operation were analyzed.
3. Taking 6 * 6 and 20 * 40 as examples to illustrate the specific implementation steps of the proactive scheduling strategy, it has certain guiding significance for the smart workshop scheduling supported by IoMT.

Aiming to reduce the delay in makespan, local adjustment and right-shift strategy were proposed to implement the proactive job-shop scheduling. In the actual production process, there are other stochastic events such as equipment breakdowns, rush orders and quality accidents, which make the pre-scheduled plan cannot execute as its wish. Therefore, the next work was to combine combinatorial optimization technology and Gantt chart to mine more scheduling rules to provide a more comprehensive scheme.
